# Sural nerve involvement in experimental hypertension: morphology and morphometry in male and female normotensive Wistar-Kyoto (WKY) and spontaneously hypertensive rats (SHR)

**DOI:** 10.1186/1471-2202-13-24

**Published:** 2012-03-02

**Authors:** Luciana Sayuri Sanada, Andréa Lurdes da Rocha Kalil, Marcelo Rodrigo Tavares, Milena Cardoso Maia Neubern, Helio Cesar Salgado, Valéria Paula Sassoli Fazan

**Affiliations:** 1 Department of Neuroscience and Behavioral Neurosciences, University of São Paulo, Av. Bandeirantes 3900, Monte Alegre, Ribeirão Preto, São Paulo 14049-900, Brazil; 2 Department of Surgery and Anatomy, University of São Paulo, Av. Bandeirantes 3900, Monte Alegre, Ribeirão Preto, São Paulo 14049-900, Brazil; 3 Department of Physiology, School of Medicine of Ribeirão Preto, University of São Paulo, Av. Bandeirantes 3900, Monte Alegre, Ribeirão Preto, São Paulo 14049-900, Brazil

**Keywords:** Sural nerve, Morphometry, Myelinated fibers, Spontaneously Hypertensive Rat, Gender differences

## Abstract

**Background:**

The sural nerve has been widely investigated in experimental models of neuropathies but information about its involvement in hypertension was not yet explored. The aim of the present study was to compare the morphological and morphometric aspects of different segments of the sural nerve in male and female spontaneously hypertensive (SHR) and normotensive Wistar-Kyoto (WKY) rats. Rats aged 20 weeks (N = 6 in each group) were investigated. After arterial pressure and heart rate recordings in anesthetized animals, right and left sural nerves were removed and prepared for epoxy resin embedding and light microscopy. Morphometric analysis was performed with the aid of computer software, and took into consideration the fascicle area and diameter, as well as myelinated fiber number, density, area and diameter.

**Results:**

Significant differences were observed for the myelinated fiber number and density, comparing different genders of WKY and SHR. Also, significant differences for the morphological (thickening of the endoneural blood vessel walls and lumen reduction) and morphometric (myelinated fibers diameter and G ratio) parameters of myelinated fibers were identified. Morphological exam of the myelinated fibers suggested the presence of a neuropathy due to hypertension in both SHR genders.

**Conclusions:**

These results indicate that hypertension altered important morphometric parameters related to nerve conduction of sural nerve in hypertensive animals. Moreover the comparison between males and females of WKY and SHR allows the conclusion that the morphological and morphometric parameters of sural nerve are not gender related. The morphometric approach confirmed the presence of neuropathy, mainly associated to the small myelinated fibers. In conclusion, the present study collected evidences that the high blood pressure in SHR is affecting the sural nerve myelinated fibers.

## Background

The sural nerve in rats is one of the most distal sensory nerves to the foot and contains a small percentage of motor fibers for the intrinsic muscles of the lateral fingers. It is the most common nerve used for investigating neuropathies, not only in humans but also in experimental models. Nevertheless, its morphology was not yet completely investigated in normotensive Wistar-Kyoto rats (WKY), as well as in spontaneously hypertensive rats (SHR). Spontaneously hypertensive rats (SHR), first inbred from WKY, are considered a good experimental model of human essential hypertension [1,2]. Hypertension is a main risk factor for stroke and vascular dementia and may cause important changes to the cerebrovascular tree, turning the brain more susceptible to infarcts, microaneurysms and ischemia [3]. In spite of the well documented influence of hypertension on the brain, data on the sensitivity of peripheral nerves in hypertension is scarce [3-7].

Another important issue that is under investigation by several authors is the influence of gender in the alterations, injuries and recovery after a lesion of the nervous system. Studies have demonstrated that differences between genders influence the re-myelination, pain sensitization, neural regulation of the vascular function, *in vitro *axonal growth, and conduction velocity of nerves [8-10]. However, few reports deal with the influence of gender and hypertension in morphological and/or morphometric differences in the peripheral nerves of mammals [6].

The aim of the present study was to describe morphological and morphometric parameters of sural nerves fascicles and myelinated fibers, in adult male and female normotensive WKY rats. Also, sural nerve alterations in adult age matched SHR, with well-established hypertension were investigated and the influence of gender in these alterations is described.

## Methods

Experiments were performed in SHR and WKY, born and raised in the animal care facility of the Department of Neuroscience and Behavioral Neurosciences, School of Medicine of Ribeirão Preto, in a controlled environment (room temperature between 21-23°C, air humidity between 40 and 70% and dark/light cycle of 12 h), housed in plastic cages (3-4 animals to a cage) with free access to tap water and rat chow throughout the experiments. All experimental procedures adhered to The Guide for the Care and Use of Laboratory Animals prepared by the National Academy of Sciences and published by the National Institutes of Health (Copyright ^© ^1996 by the National Academy of Sciences), and were approved by the Institutional Ethics Committee for Animal Research (CETEA--Comitê de Ética em Experimentação Animal, protocol number 184/2005). Effort was made to minimize the number of animals used.

Male (N = 6) and female (N = 6) SHR and WKY, brothers and sisters, 20 weeks old were anesthetized with sodium thiopental (Thionembutal, 40 mg kg, i.p.) and a catheter was inserted into the femoral artery for measurement of arterial pressure (AP). Recordings of the systolic (SAP), diastolic (DAP), mean arterial pressure (MAP) and heart rate (HR) were performed as described elsewhere [11,12]. After the recordings, rats were perfused through the left ventricle first with a 0.05 M phosphate-buffered saline solution, pH 7.4 and then with a 2.5% glutaraldehyde solution in 0.1 M cacodylate buffer, pH 7.2. Both right and left sural nerves, from their origin in the hip (5-7 mm distal to the greater trochanter) through their distal branching at the lateral malleolus level, were carefully dissected without stretching, removed in one piece and placed in the fixative solution for an additional 12 hour. They were washed in cacodylate buffer, pH 7.2, and proximal (close to the origin) and distal (close to terminal branching) segments (of approximately 3 mm each) were cut and processed for epoxy resin embedding (PolyBed 812^®^, Polysciences Inc., Warrington, PA, USA) as described elsewhere [13-15]. Samples of all four experimental groups were histologically processed at once so that they were submitted to absolutely the same experimental conditions throughout the experiments.

Semithin (0.2-0.3 μm thick) transverse sections of the fascicles were stained with 1% toluidine blue and examined with the aid of an Axiophot II photomicroscope (Carl Zeiss, Jena, Germany). The images were sent via a digital camera (TK- 1270, JVC, Victor Company of Japan Ltd, Tokyo, Japan) to an IBM/PC where they were digitized. For the study of myelinated fibers, the endoneural space was observed with an optical set including an oil immersion lens (100 ×), optovar (1.6 ×), camera (0.5 ×) and an 8 × computerized magnification, which provided images with good resolution for morphometry.

The endoneural space was fully scanned without overlap of the microscopic fields, using an automatic motorized stage (Carl Zeiss, Jena, Germany). Scannings generated 9 to 17 microscopic fields of 640 × 470 pixels, which were used to count and automatically measure the myelinated fibers and their respective axons. Fibers at the upper and left edges of the microscopic fields were counted whereas those at the lower and right edges were not counted ("forbidden line") in order to avoid counting the same fiber twice. All myelinated fibers present in the endoneural space were counted. Morphometric parameters of the fascicles and myelinated fibers of sural nerve segments were obtained as described previously for other nerves [7,14-16]. Briefly, the total number of myelinated fibers and the total number of Schwann cell nuclei present in each fascicle transverse section were counted. The area and lesser diameter of each fascicle (excluding the perineurium) as well as each myelinated fiber (defined by the axon and its respective myelin sheath excluding the Schwann cell nucleus when present) and respective axon (fiber excluding the myelin sheath) were measured with image analysis software (KS 400, Kontron 2.0, Eching Bei München, Germany). The lesser diameter better represents the diameter of a non-circular fascicle and fiber [13,17,18]. The area values are directly calculated by the software ("filled area" function), not calculated by from perimeter value. The percentage of the total cross-sectional area of the endoneural space occupied by the myelinated fibers was calculated, and hereafter referred as the percentage of occupancy of the myelinated fibers [6,7,13-15]. The myelinated fibers and Schwann cell nuclei densities were calculated. For myelinated fibers, both axonal diameter and total fiber diameter were automatically measured. The ratio between the two diameters, the g ratio (which indicates the degree of myelination), was obtained [19,20]. Myelin sheath area was calculated for each myelinated fiber measured. For diameter measuring, fiber and axon forms are taken into account by "circularity index" calculation for each fiber. When fibers present index higher than 0.45, they are measured. Thus, fibers that are too oblong or irregular are automatically discharged. Histograms of population distribution of myelinated fibers and axons, separated into class intervals increasing by 1.0 μm were constructed. Histograms of the g ratio distribution separated into class intervals increasing by 0.1 were also prepared. The investigators were blind to group identities throughout the experiments.

Morphometric data were tested for normal distribution by the Kolmogorov-Smirnov normality test followed by the Levene test for variance equivalence. If data presented a normal distribution and equivalent variance, comparisons were made between proximal and distal segments in the same group by paired Student's t-test. Otherwise, comparisons were made by Wilcoxon's non-parametric test for paired samples. For comparisons between right and left segments in the same group, normally distributed data were tested using the unpaired Student's t-test. Alternatively, comparisons were made by the Mann-Whitney non-parametric test. Comparisons between groups were made by one-way analysis of variance (ANOVA) followed by Holm-Sidak post hoc test. Comparisons between histograms were made by one-way analysis of variance (ANOVA) on Ranks provided that the distributions did not pass the normality test. For all applied statistical tests, differences were considered significant when p < 0.05. Data are presented as mean ± standard error of the mean (SEM).

## Results

### Body weight and arterial blood pressure

Body weight and hemodynamic parameters of male and female WKY and SHR, are shown in Table 1. Male WKY and SHR showed greater body weight when compared to females from the same strain. Also, male and female SHR showed higher systolic, diastolic and mean arterial pressure, but lower body weight, as compared to WKY of same gender. Male SHR presented higher systolic blood pressure as compared to female SHR. No hemodynamic differences were observed between male and female WKY.

**Table 1:** Body weight and physiological data of male and female SHR and WKY

	Female WKY	Male WKY	Female SHR	Male SHR
Body Weight (g)	218.67 ± 14.33	388.25 ± 20.03*	194.33 ± 13.32^#^	325.00 ± 16.11*^#^
SAP (mmHg)	174.33 ± 17.57	153.00 ± 8.31	208.22 ± 11.31^#^	241.63 ± 18.08*^#^
DAP (mmHg)	117.33 ± 13.21	118.75 ± 6.43	156.56 ± 9.19^#^	170.24 ± 22.19^#^
MAP (mmHg)	143.67 ± 15.42	136.50 ± 6.61	180.06 ± 17.90^#^	200.75 ± 18.18^#^

Data expressed as mean ± standard error of mean (SEM). *Indicates significant differences between male and female (unpaired Student's t-test, p < 0,05); ^#^Indicates significant differences between WKY e SHR (unpaired Student's t-test, p < 0,05). SAP = systolic arterial pressure, DAP = diastolic arterial pressure, MAP = mean arterial pressure

### Morphological aspects

**Figure 1:**
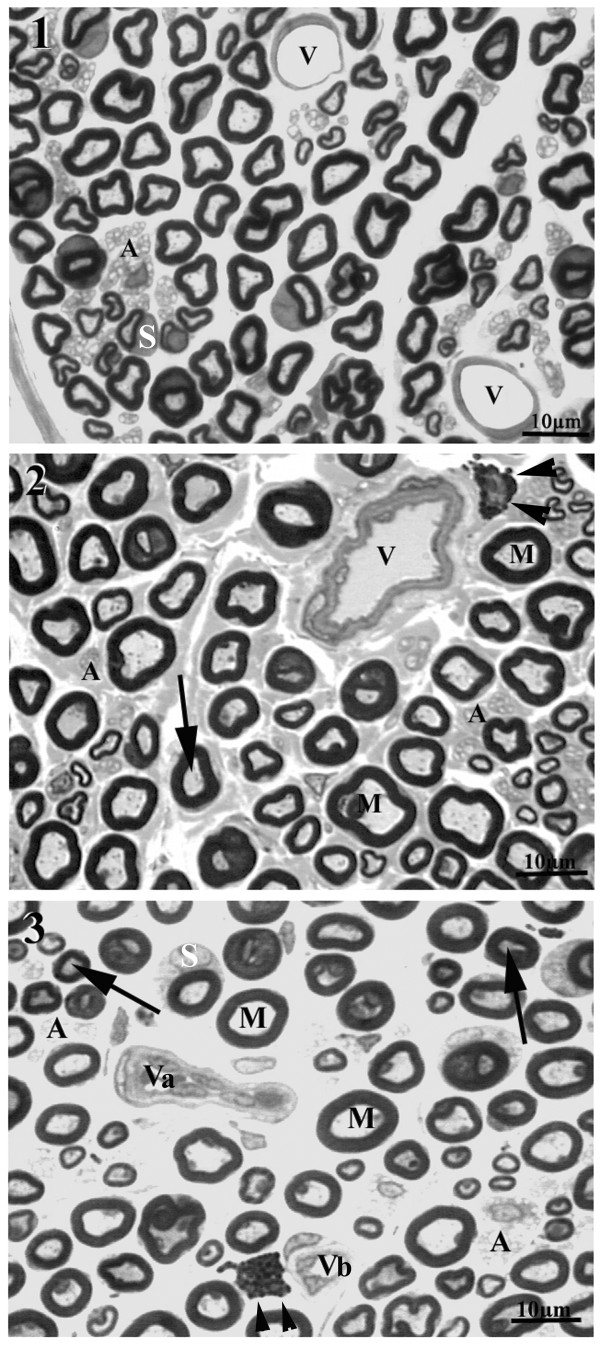
**Representative semithin cross section of the sural nerve of young (20 weeks old) male WKY (1) and male SHR (2 and 3), showing typical endoneural structures**. Large (M) and small myelinated fibers as unmyelinated (A) fibers are present in the endoneural space. Mast cells are indicated by arrowheads and Schwann cell nuclei are indicated by S. Note the presence of normal endoneural vessels (V) in the WKY nerve while in SHR thickening of the wall (in 2), collapsed vessels (Va) and vessels with endoneural hyperplasia (Vb) were common. Arrows indicate large axons with atrophy. Toluidine blue stained. Bar = 10 μm.

**Figure 2:**
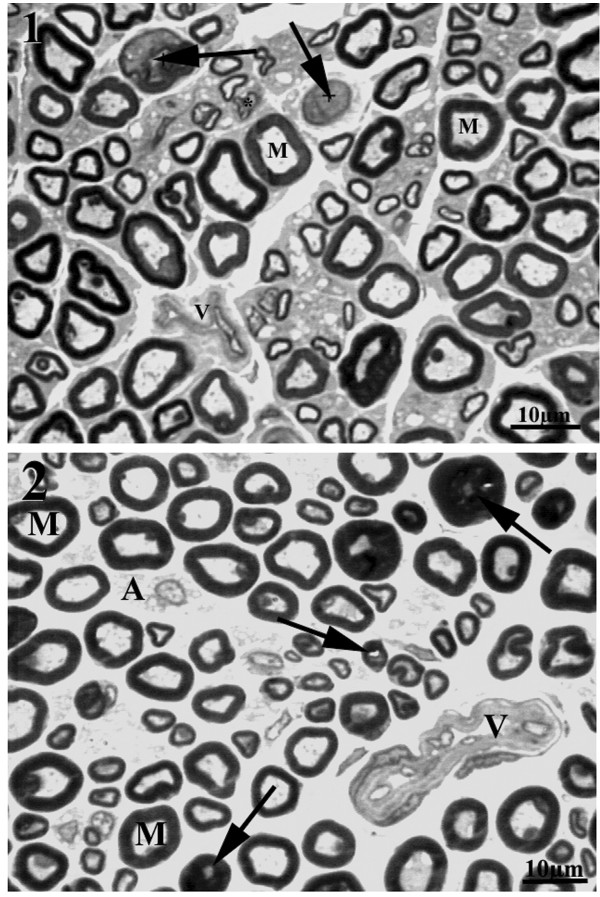
**Representative semithin cross section of the sural nerve of young (20 weeks old) female (1) and male SHR (2)**. Note the presence of infolded myelin sheath and myelin splitting. Arrows point to Wallerian degenerations. V indicates collapsed endoneural vessels and M indicates large myelinated fibers with normal aspect. Toluidine blue stained. Bar = 10 μm.

All sural nerves included in this study showed good preservation of structures. One or more fascicles were observed in the cross sections and general morphological characteristics of the sural nerve fascicles of WKY and SHR were similar to those described for female Wistar rats [13,14]. Also, the main components of the sural nerves did not differ from those of other peripheral nerves. Most proximal segments of the sural nerves in male and female WKY and SHR showed a single fascicle, while two or more fascicles were present on the distal segments. No endoneural morphological differences were observed between proximal and distal segments from the same side, and between the same segment (proximal or distal) from different sides, for all groups. However, the comparison between strains indicated that the sural nerves from male and female SHR showed a larger number of collapsed blood vessels (thickening of the walls, and absence of lumen) and/or vessels with thickening of the wall (Figure 1). In addition, myelinated fibers with degenerative signs were present in female and male SHR. These signs consisted of the presence of contorted and infolded myelin sheaths and myelin loops and splitting. Large myelinated fibers with thin myelin sheaths were observed, while grossly swollen demyelinated axons were occasionally present. Wallerian degeneration was also shown. Small myelinated fibers with signs of axonal atrophy were present; this alteration appeared to be more frequent in males than females SHR (Figure 2).

### Fascicle morphometry

Data are shown in Table 2. The area and diameter of the distal segments of the sural nerves were significantly smaller than those from the proximal segments in female rats of both strains. The same observation was made for the myelinated fibers number. Nerves from males tended to be larger than from females on both strains. The comparison between strains revealed that SHR nerves were larger (area and diameter) than WKY in both genders, with a significant difference on the fascicle diameter of the distal segments. However, male and female SHR presented a smaller number and density of myelinated fibers and Schwann cell nuclei and a smaller capillary percentage of occupancy than WKY, especially on the distal segments.

**Table 2:** Morphometric parameters of proximal and distal segments of the sural nerve fascicles of male and female WKY and SH

	Female WKY	Male WKY	Female SHR	Male SHR	Female WKY	Male WKY	Female SHR	Male SHR
	**Proximal Segments**					**Distal Segments**		

FA (μm^2^)	49096 ± 3140	52479 ± 3052	51596 ± 2484	54504 ± 3062	38162 ± 2635*	41188 ± 3998	44956 ± 4812*	50144 ± 4272
Fascicle Ø (μm)	193 ± 16	181 ± 22	205 ± 14	184 ± 15	89 ± 4*	113 ± 20	146 ± 9*^#^	155 ± 15^#^
MF number	871 ± 25	831 ± 47	889 ± 36	884 ± 33	746 ± 47*	725 ± 53	690 ± 47*	688 ± 45
MFD (fibers/μm^2^)	18469 ± 1072	15986 ± 724	17454 ± 715	16541 ± 647	19849 ± 821	18119 ± 1059	16370 ± 972^#^	14770 ± 1586^#^
SCN number	40 ± 3	42 ± 5	35 ± 4	35 ± 2	33 ± 2	35 ± 3	35 ± 5	27 ± 2
SCND (nuclei/μm^2^)	843 ± 58	810 ± 100	731 ± 84	666 ± 48	906 ± 83	889 ± 60	800 ± 68^#^	576 ± 78^#^
MF occupancy (%)	38.79 ± 1.46	36.90 ± 1.65	40.10 ± 1.82	41.30 ± 2.96	40.15 ± 1.56	39.80 ± 1.99	35.75 ± 1.62	35.09 ± 3.01
CP occupancy (%)	0.96 ± 0.26	0.90 ± 0.22	0.40 ± 0.13	0.55 ± 0.09	0.71 ± 0.19	0.69 ± 0.13	0.18 ± 0.06#	0.28 ± 0.12#

Data expressed as mean ± standard error of mean (SEM). FA = fascicle area, Ø = diameter, MF = myelinated fiber, MFD = myelinated fiber density, SCN = Schwann cell nuclei, SCND = Schwann cell nuclei density, CP = capillary. * indicates differences between proximal and distal segments (paired Student's t-test p < 0.05); # indicates differences between WKY and SHR (unpaired Student's t-test, p < 0.05)

### Myelinated fiber morphometry

**Figure 3:**
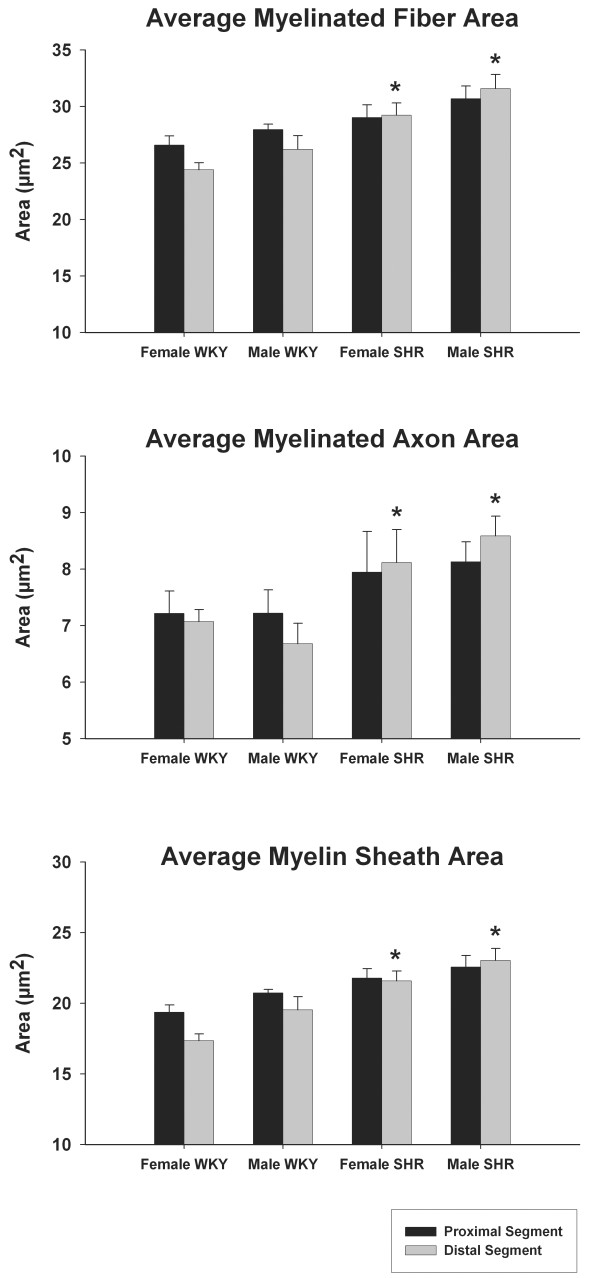
**Average myelinated fiber and respective axon and myelin sheath areas of the right sural nerve of young (20 weeks old) male and female SHR and WKY**. Note the trend towards smaller values on the distal segments in WKY nerves and an inversion of this trend in SHR. * indicates difference compared to WKY (unpaired Student's t-test). No differences between sides were detected (unpaired Student's t-test).

Myelinated fibers and respective axons and myelin sheath average area in right segments of male and female WKY and SHR sural nerves are illustrated in Figure 3. No morphometric differences were observed among segments and sides in all groups and between genders in the same animal strain. A tendency toward smaller values on the distal segments was observed in WKY while for SHR the opposite situation was noticed. The comparison between different strains showed statistical significance for distal segments in both genders, being the average area values larger in SHR (Figure 3).

**Figure 4:**
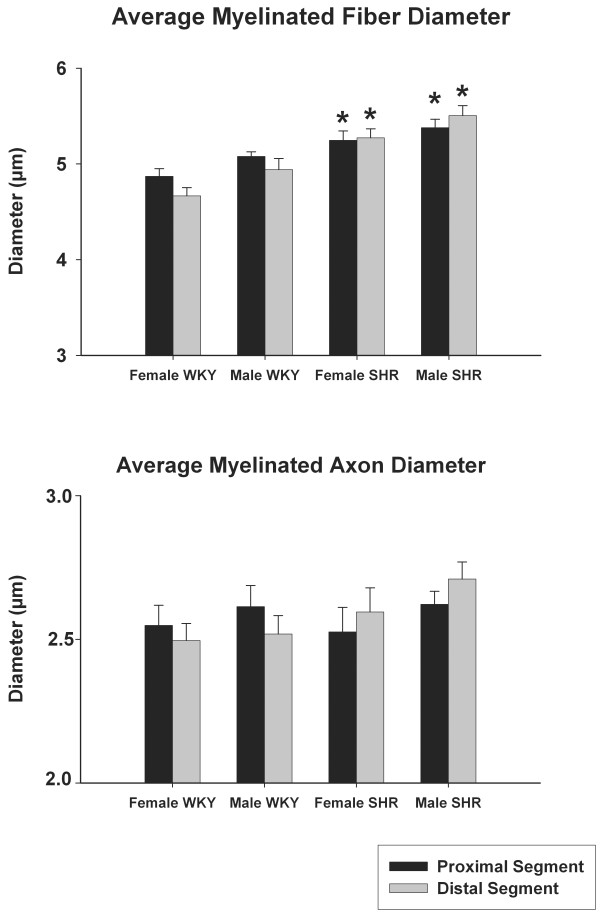
**Average myelinated fiber and respective axon diameters of the right sural nerve of young (20 weeks old) male and female SHR and WKY**. Note the trend towards smaller values on the distal segments in WKY nerves and an inversion of this trend in SHR. * indicates difference compared to WKY (unpaired Student's t-test). No differences between sides were detected (unpaired Student's t-test).

Myelinated fibers and their respective axon diameter of the right sural nerves are shown in Figure 4. No morphometric differences were observed between segments and sides in all groups and between males and females in the same strain. Again, a tendency towards smaller values on the distal segments was observed in WKY while for SHR the opposite situation was evident. The comparison between strains revealed a larger fiber diameter for male and female SHR on proximal and distal segments. No differences were observed for the axon diameter (Figure 4).

**Figure 5:**
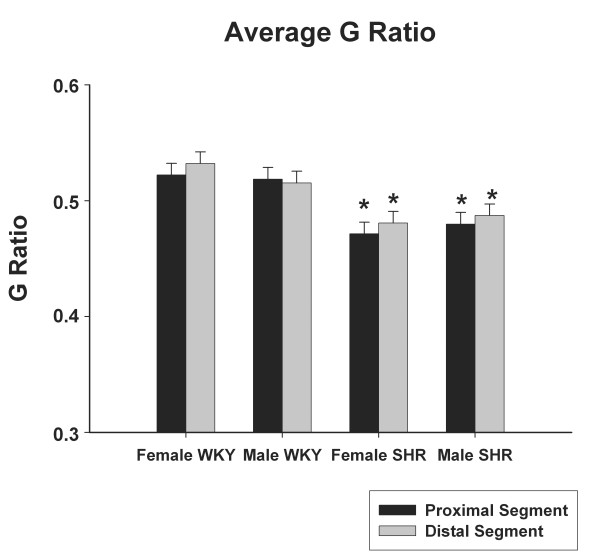
**Average myelinated fiber G ratio of the right sural nerve of young (20 weeks old) male and female SHR and WKY**. * indicates difference compared to WKY (unpaired Student's t-test). No differences between sides were detected (unpaired Student's t-test).

The average G ratio for proximal and distal segments of the right sural nerves in male and female WKY and SHR are represented in Figure 5. No differences were observed among segments and sides in all groups or between genders in the same strain. Male and female SHR showed significantly smaller G ratio on both segments, compared to WKY, in both genders.

**Figure 6:**
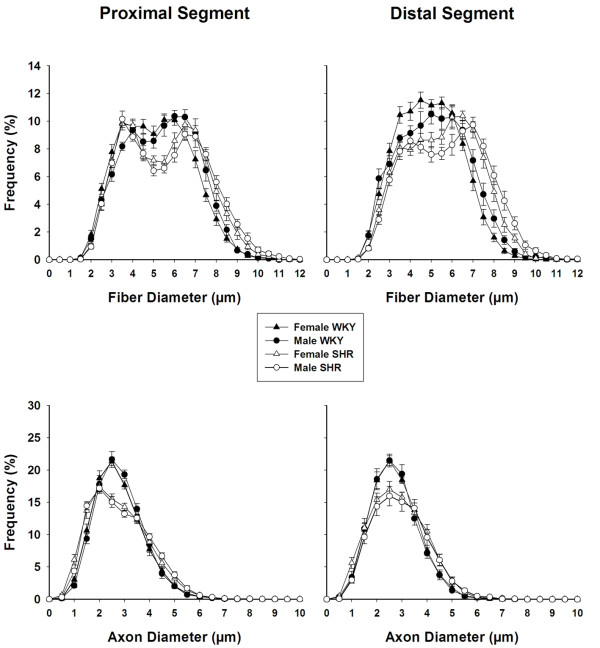
**Size distribution of myelinated fibers (upper panels) and respective axons (lower panels) of proximal (left panels) and distal (right panels) segments, of the right sural nerve of young (20 weeks old) male and female SHR and WKY**. Note that myelinated fibers distributions are bimodal while axon distributions are unimodal. Note also a reduction of the frequency of the smaller fibers and axons in SHR nerves. No differences were observed between sides (One Way Analysis of Variance (ANOVA) on Ranks test).

Distributions of myelinated fibers and respective axons for proximal and distal segments of the right sural nerves in male and female WKY and SHR are shown in Figure 6. Myelinated fiber diameter ranged between 1.50 and 12.00 μm, and was distributed bimodally, with peaks of frequency between 3.00-4.00 and 6.00-7.00 μm in proximal segments. In distal segments, peaks of frequency were not so well defined, especially in WKY, but most fibers ranged within diameters of 3.00-6.00 μm. No statistical differences were observed between segments, sides and genders of the same strain. SHR histograms were shifted to the right and a reduction on the frequency of the small myelinated fibers is observed in all segments. Myelinated axon diameter ranged between 0.50 and 8.50 μm, with an unimodal distribution (Figure 6). Peaks of frequency between 2.00-3.00 μm were observed for all groups but SHR nerves showed a clear reduction of the frequency of small axons, compared to WKY. No statistical differences among segments, sides and genders of the same strain were observed for the myelinated axon distributions.

## Discussion

### Body weight and hemodynamic data

Our results indicated that male SHR and WKY are heavier than females of the same strain and also that WKY are heavier than SHR from both genders. Differences between the body weight of male and female SHR and WKY, as well as the differences between strains observed in the present study were similar to those described in the literature [6,7,21-27].

As expected, SAP levels were significantly different between SHR genders, being higher in males and SAP, DAP and MAP were significantly higher in both SHR genders, compared to WKY. Overall, these data are in line with previous findings from the literature [6,7,21-28].

### Morphological and morphometric aspects of sural nerve in SHR and WKY

The present study provides a complete morphometric assessment of the sural nerve fascicles and myelinated fibers in male and female SHR, as compared to normotensive WKY rats. Morphologic characteristics of sural nerve in WKY and SHR strains are described for the first time. Morphological alterations of myelinated fibers and endoneural blood vessels were found in the sural nerves of male and female SHR, while the nerves of WKY were morphologically similar to the sural nerves from other rat strains [13,14].

Sabbatini et al. [29,30] showed luminal narrowing and increase wall-to-lumen ratio of small intrafascicular arteries in SHR sciatic nerve. This is in accordance to our observation in the sural nerve. Nevertheless, those authors [29,30] did not investigate the nerve fibers. Our results showed morphological alterations in both large and small myelinated fibers. Alterations of the myelin sheath were more evident on large fibers while the small fibers showed morphological signs of atrophy. Also, a reduction of myelinated fiber number was evident in SHR.

Tomassoni et al. [3] investigated the myelinated fibers of sciatic nerves in SHR. They found reduction of the myelin area on large myelinated fibers with no reduction of the number of fibers. Nevertheless, they used thick paraffin sections for the microanatomical and morphometric analysis. In the present study, semithin sections were used, providing a better resolution, especially to investigate the thin myelinated fibers, that can be easily neglected when thick section are analyzed because they are more difficult to stain [31-33]. The reduction of fiber number observed in the present study is mainly due to a small fiber loss, confirmed morphometricaly by a reduction of the small fiber class intervals (Figure 6), with no important shift of the histograms. Also, average myelinated fiber and axon areas are larger on SHR, showing that the small fiber loss shifted the average values towards larger ones.

In neuropathies, it is well known that degenerative changes occur first in the distal portion of the long fibers [34-36]. The average morphometric parameters of the sural nerve fibers in WKY showed a trend towards smaller values on distal segments, compatible to the observations from others [13,14]. However, in the SHR nerves, this trend was inverted (Figures 3 and 4), indicating that distal segments were more affected by hypertension than the proximal segments.

Experimental models of arterial infarct suggested that small nerve fibers are more vulnerable to ischemia than the large nerve fibers [37]. Giannini and Dyck [38] described that thickening of the wall in blood vessels may be the responsible for the peripheral neuropathy in diabetes. Likewise, Fazan et al. [39] demonstrated a small fiber neuropathy associated to a damage of the endoneural vessels, similar to the observation of the present study. Our observations from SHR's nerves are in line with these findings, particularly because the alterations of the small myelinated fibers were associated to lesions on the endoneural vessels, which were severe enough to cause endoneural ischemia.

Confirming the alterations in SHR, our data show a larger myelinated fiber G ratio in WKY as compared to SHR (Figure 6). This data suggest that hypertension may be responsible for an axonal atrophy in sural nerve of male and female SHR, with no differences between genders, despite the significant difference on SAP. Similar to the present study, Fazan et al. [4,5] demonstrated that hypertension caused a decrease in axon size, myelin sheath and unmyelinated fiber number in the aortic depressor nerve of SHR.

Tomassoni et al. [3] demonstrated that the increase in arterial blood pressure was responsible for morphological and morphometric alterations in myelinated fibers of sciatic nerve, that correlated to a decrease of the conduction velocity of this nerve. The present study showed reduced average G ratio and a shift of the histograms to the left in male and female SHR. These results suggest that the myelinated fibers of SHR sural nerves might have a reduced conduction velocity probably due to an axonal atrophy, as observed for the sciatic nerve [3].

Most of experimental studies involving sural nerve morphology have been carried out on male rats [40-42] whereas little information is available from female rats [13,14,43]. Nevertheless, sex-related differences in the outcome of nervous system injuries and disorders have been an important issue in the last decades. The present study showed no morphological or morphometric differences in the sural nerves between male and female rats for both WKY and SHR, despite differences in the body weight and SAP among genders.

## Conclusions

In the present study, morphological alterations were evident in endoneural blood vessels in male and female SHR. Morphological exam of the myelinated fibers suggested the presence of a neuropathy due to hypertension in both genders of SHR. The sophisticated morphometric approach used in the present study confirmed the presence of neuropathy, mainly associated to the small myelinated fibers. In conclusion, the present study collected evidences that the high blood pressure in SHR is affecting the sural nerve myelinated fibers. Moreover, the absence of morphologic and morphometric differences in the peripheral nervous system of male and female normotensive rats validate those morphological studies where males and females were used indistinctly.

## Authors' contributions

LSS: responsible for acquisition of data, analysis and interpretation. ALdRK: responsible for acquisition of data and analysis. MRT: responsible for acquisition of data and analysis. MCMN: responsible for acquisition of data. HCS: responsible for critically revising the manuscript. VPSF: responsible for conception and design, interpretation of data, drafting the manuscript and for given final approval of the version to be published.
